# Decreased Levels of Vitamin D in Bipolar Patients

**DOI:** 10.3390/life13040883

**Published:** 2023-03-27

**Authors:** Donatella Marazziti, Paola Mangiapane, Manuel Glauco Carbone, Florinda Morana, Alessandro Arone, Lucia Massa, Stefania Palermo, Miriam Violi, Giovanni Bertini, Leonardo Massoni, Sara Fantasia, Andrea Pozza, Federico Mucci, Benedetto Morana

**Affiliations:** 1 Department of Clinical and Experimental Medicine, University of Pisa, 56126 Pisa, Italy; 2 Department of Medicine and Surgery, Saint Camillus International University of Health and Medical Sciences—UniCamillus, 00131 Rome, Italy; 3 Casa di Cura Morana, 91025 Marsala, Italy; 4 Department of Medicine and Surgery, Division of Psychiatry, University of Insubria, 21100 Varese, Italy; 5 Department of Medical Science, Surgery and Neuroscience, University of Siena, 53100 Siena, Italy; 6 Department of Biotechnology, Chemistry and Pharmacology, University of Siena, 53100 Siena, Italy

**Keywords:** vitamin D, biomarkers, mood disorders, bipolar disorders, treatment resistance, augmentation strategies

## Abstract

Recently, vitamin D is considered a pleiotropic hormone, and as such, it has also become a topic of renewed interest in neuropsychiatry for its proposed role in the aetiology and pathophysiology of different psychiatric conditions, including mood disorders (MDs). This seems particularly crucial while considering the relatively high and often neglected prevalence of hypovitaminosis D in the general population and in specific groups, such as patients suffering from the most common type of MDs, which are major depression (MDD) and bipolar disorders (BDs). Therefore, in view of the controversial literature and findings on this topic and its potential therapeutic implications, the present study aimed at evaluating vitamin D levels in the plasma of a sample of inpatients fulfilling the DSM-5 criteria for mood episodes within BDs. The clinical picture was assessed by means of specific rating scales. The results showed that the vitamin D levels (mean ± SD, nM/L) of the bipolar patients of our sample were significantly lower (14.58 ± 11.27 nmol/L) than the normative values (>30 nmol/L). Eleven patients had sufficient values and only 4 had optimal, while 19 showed insufficient, 18 critical, and 17 severely critical levels. No differences emerged according to different socio-demographic or clinical features. In our opinion, the present findings strengthen previous research highlighting decreased vitamin D levels in bipolar patients and support the role of this pleiotropic hormone in BDs. Nevertheless, further studies should follow to corroborate the data of this preliminary study and to address the potential benefits of vitamin D supplementation in the treatment of MDs.

## 1. Introduction

Mood disorders (MDs) show a high lifetime prevalence in the general population and represent a leading cause of disability worldwide [1]. As reported by the World Health Organization, about 350 million people of all ages suffer from MDs globally. Indeed, these disorders cause marked impairment in social and occupational functioning, resulting in a high burden for the individual and society [2]. Mood disorders represent a wide group of psychiatric conditions that, according to the Diagnostic and Statistical Manual of Mental Disorders fifth edition (DSM-5) [3] criteria, are distinguished into major depression (MDD), bipolar disorders (BDs), cyclothymic disorder, dysthymia, disruptive mood dysregulation disorder, and premenstrual dysphoric disorder. Specifically, if depressive episodes are present in both MDD and BDs, the diagnosis of BD of type 1 (BDI) or BD of type 2 (BDII) is made on the basis of the presence of, respectively, manic or hypomanic episodes.

Currently, it is agreed that the aetiology and pathophysiology of BDs are complex and multifaceted, as probably they involve the intertwining between individual vulnerability that might also be based on genetics, epigenetics, neurotransmitters, immune and central nervous (CNS) systems, and environmental factors [4,5,6,7,8,9,10,11].

Recently, converging data highlighted that nutritional/dieting factors may show relevant relationships with the aetiology, evolution, prevention, and, possibly treatment of mental disorders [12]. Given their pleiotropic functions encompassing those recently explored in the CNS, the possible roles of some vitamins have also been investigated in MDs. Data are already available suggesting that low levels of vitamin B12 and folate might represent an increased risk of developing depression [13,14,15,16]. Currently, much interest in the scientific community has also been directed toward vitamin D [17,18,19]. Since its discovery, it is well known and agreed that vitamin D is important in the maintenance of the bone structure and calcium/phosphorus homeostasis [20]. However, there is now compelling evidence that it is critical in regulating cell proliferation and differentiation [20], apoptosis [21], and even a variety of modulatory activities of the immune system [22]. Furthermore, vitamin D plays a manifold role in several functions of the CNS. Animal data show that it regulates brain development during early life [23] and promotes axogenesis [24] and several neurotrophic factors, especially the nerve growth factor (NGF) [25,26]. Vitamin D may also act as a neuroprotective factor, possibly through different mechanisms to be still elucidated, as it may decrease toxicity through the blockade of calcium influx [27] or inhibition of nitric oxide production [28].

In spite of the increasing amount of data on the high prevalence of different degrees of hypovitaminosis D in the general population worldwide, this problem is largely underestimated, with the disappointing outcome that the achievement of the recommended vitamin D status is an objective far from being reached even in several most-advanced Western countries [29].

Albeit increasing, scattered findings would indicate that patients suffering from different psychopathological conditions, such as psychotic disorders [30], anxiety disorders [31], eating disorders [32], obsessive-compulsive disorder (OCD) [33], post-traumatic stress disorder (PTSD) [34], and autism spectrum disorders [35] may show vitamin D deficiency.

The same is true for MDs, as the overall findings are intriguing but still controversial, mainly for the paucity of available evidence. A link between depressive symptoms and lower vitamin D levels was reported in different samples of adult depressed patients [36,37,38,39]. Treatment with antidepressants would seem to improve vitamin D levels, although they continue to be insufficient [40]. Interestingly, the deficiency of vitamin D was reported to be more common amongst depressed females as compared with depressed males [41].

In BDs, vitamin D deficiency has been reported in acute manic episodes [42] and in psychotic patients [43]. In the majority of the studies, the average values of vitamin D in BD samples were under the threshold of vitamin D deficiency. A cross-sectional study performed on 100 euthymic patients with BDs, showed a high vitamin D deficiency prevalence in this sample and identified an interaction between vitamin D and cognitive domains, an effect that was modified by age. Indeed, the age-modified interaction of vitamin D was associated with composite neurocognitive scores and verbal fluency in both age groups and with the processing speed domain in the younger group [44].

On the other hand, most of the studies reported no significant differences in vitamin D levels between patients with BDs and other psychiatric disorders, such as depression, psychosis, obsessive-compulsive disorder (OCD), and dementia [33,45,46]. Again, a high prevalence of vitamin D deficiency was found in a group of elderly patients with a wide range of psychiatric diagnoses. After adjusting for age, gender, season, body mass index, and smoking, vitamin D deficiency was still associated with the patient’s status. Therefore, although an association between vitamin D levels and clinical symptomatology was observed in some bipolar patients, this biomarker cannot be considered specific to BDs, but a common characteristic shared with other psychiatric disorders and perhaps related to specific symptoms, symptom clusters/dimensions [36,37,38,39,40,41,43,45], or severity of illness [33].

In spite of the evident, albeit controversial, findings existing in the literature, the hypothesis that vitamin D supplementation, a quite easy and tolerated treatment, might represent a useful therapeutic strategy to boost the treatment of both BDs and MDD patients is still an intriguing and debated topic. In one study, an eight-week supplementation with vitamin D3 could improve the symptoms of a manic episode [47]. Again, the eight-week supplementation with 50,000 IU cholecalciferol/2 weeks decreased depression severity [48] and ameliorated the overall quality of life and illness severity [49], with women presenting a more significant improvement than men [50]. Intervention with B vitamins and/or vitamin D was also shown to represent an effective and well-tolerated adjuvant strategy for improving the symptoms of depression and anxiety, according to the patient’s clinical status and nutritional biomarkers [31]. A large meta-analysis led to the conclusion that such a therapeutic strategy might moderately decrease depressive symptoms [51]. Further, a review of the literature, including 10 published studies, revealed that vitamin D supplementation might improve both depressive and manic symptoms of bipolar patients [45].

On the contrary, a double-blind placebo-controlled trial did not replicate these results [52], as also shown in a recent review and meta-analyses [53,54].

Given the limited and heterogeneous information currently available, together with the potential therapeutic impact, the aim of the present study was at evaluating vitamin D levels in a group of hospitalized patients suffering from BDs, well characterized from the clinical point of view. In addition, we assessed the possible existence of correlations between the biomarker and patients’ characteristics.

## 2. Materials and Methods

Sixty-nine inpatients of both sexes (40 women and 29 men, mean age: 45.09 ± 14.42 years) were recruited at the “Casa di cura Morana” (Marsala, Sicily, Italy) in the month’s March–June 2021. All patients had been hospitalized for BDs, according to DSM-5 criteria, given the severity of their mood episodes. They belonged to the same geographical area, with similar life habits and Mediterranean diet. The clinical examination and routine blood and urine tests excluded major medical diseases.

### 2.1. Assessment Scales

The rating scales used to assess the quality and severity of the clinical characteristics were the following: Mini International Neuropsychiatric Interview (MINI) [55], Hamilton Rating Scale for Depression (HRSD) [56], Young Mania Rating Scale (YMRS) [57], and Clinical Global Impression-Severity scale (CGI) [58].

### 2.2. Mini International Neuropsychiatric Interview (MINI)

The MINI is a structured clinical interview widely used by clinicians to diagnose the most common psychiatric disorders, according to DSM criteria. It is a rapid, sensitive, reliable instrument to be used easily in clinical practice [55].

### 2.3. Hamilton Rating Scale for Depression (HRSD)

The 21-item HRSD represents the most used tool to assess clinical depression. It is a self-rating scale that is easy and fast to complete and takes into consideration both the extent and frequency of the symptoms [56]. The severity cut-off is defined as follows: >25 severe depression; 18–24 moderate depression; 8–17 mild depression; <7 no depression.

The mean HRSD total score of the patients in the depressed phase was 26 ± 4.

### 2.4. Young Mania Rating Scale (YMRS)

The YMRS is an 11-item scale that explores the key symptoms of mania that are generally present throughout the entire clinical course (from the most modest to the most severe phases) [57].

The mean YMRS total score of the patients in the manic phase was 40 ± 5.

### 2.5. Clinical Global Impression-Severity (CGI-s)

The CGI-S is a questionnaire to rate the severity of the patient’s illness at the time of the visit. The scale is endowed with three items. The ensuing scores are the following: 1. Healthy; 2. Borderline; 3. Slightly ill; 4. Moderately ill; 5. Markedly ill; 6. Severely ill; 7. Very high severity [58].

All patients showed a score between 5 and 7.

### 2.6. Statistical Analyses

All data for continuous variables were presented as ranges (min and max values) and mean ± standard deviation (SD). Frequencies (numbers) and percentages were used to express categorical variables. The normality of the distribution of the variables was assessed by the Kolmogorov-Smirnov test. The intergroup differences were determined by the one-way analysis of variance (ANOVA). The χ^2^ test (or Fisher’s exact test when appropriate) was applied to compare categorical variables. Pearson’s correlation coefficient or Spearman rank correlation was used to assess the possible correlations between different parameters and between subjects’ characteristics and biomarkers. All statistical analyses were carried out using SPSS, version 27 (IBM Corp. Released 2020. IBM SPSS Statistics for Windows, Version 27.0. Armonk, NY, USA: IBM Corp.).

### 2.7. Plasma Preparation for Vitamin D Assessment

Ten mL of venous blood was drawn from all fasting and sitting subjects between 8 and 9 a.m. The blood was then transferred to plastic tubes for vitamin D measurements by the common clinical-chemical method of competitive protein-binding (CPB) assay. All patients signed an informed written consent to collect their blood samples and to use their data on vitamin D for the aim of this study.

## 3. Results

### 3.1. Socio-Demographic and Clinical Data

The results showed that 27 patients fulfilled the DSM-5 criteria for the diagnosis of BDI, 15 of BDII, 16 of schizoaffective disorders, and 11 of MDD. Thirty-six patients were suffering from a depressive episode, 20 from mixed features, 7 from a hypomanic episode, and 6 from a manic episode, according to the MINI (Table 1).

Thirty-six patients showed psychotic symptoms: the majority of this group was suffering from BDI (3 were in a manic phase, 13 in a mixed state, and 3 in a depressive phase), 13 from schizoaffective disorder (6 were in a hypomanic phase, 3 in a manic phase, and 4 in a depressive phase), and 4 from MDD. The absence of psychiatric comorbidity was detected in 40 patients, while 18 referred substance abuse (12 cannabis, 3 psychostimulants, and 3 hallucinogens), 11 had an anxiety disorder, and 10 had mild neurodevelopmental traits. Thirty-six patients were heavy smokers (more than 20 cigarettes daily). Fifty-seven patients had at least a relative with a psychiatric disorder, and 9 had a family history of suicide.

Different psychotropic drugs had been prescribed to all patients, according to the effective requirements of the diagnosis and the polarity of the episode, as only 7 patients resulted to be drug-free at the assessment. Sixty-seven patients were taking mood stabilizers (valproic acid, lithium salts, gabapentin, pregabalin, lamotrigine, oxcarbazepine, carbamazepine), of whom 34 were taking 2, and 20 were taking 3 of these compounds. Twenty-seven patients were also treated with antidepressants (selective serotonin reuptake inhibitors [SSRIs, serotonin and norepinephrine reuptake inhibitors [SNRIs], tricyclics, mirtazapine, trazodone, bupropion). Antipsychotics (first- and second-generation antipsychotics [FGAs and SGAs]) were given to 63, benzodiazepines to 20, and anticholinergic drugs (biperiden) to 4 patients.

### 3.2. Vitamin D

**Figure 1:**
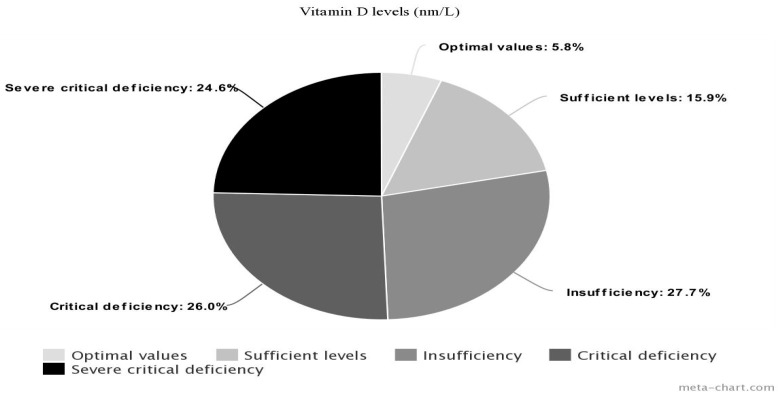
Vitamin D levels (nm/L) of bipolar inpatients.

The vitamin D (mean ± SD, nm/L) in the total sample was 14.58 ± 11.27, almost the same in the two sexes (M = 14.72 ± 12.74, F = 14.48 ± 10.24). A statistically significant difference (t = −11.36 and *p* < 0.001) was detected between patients and normative mean values (30 nm/L). Nineteen (27.54%) patients showed insufficient (12–20 nm/L: 15.70 ± 2.17) vitamin D levels, 18 (26.09%) had a critic deficiency (<12 nm/L: 9.10 ± 1.49), 17 (24.64%) had severe critic levels (<6.5 nm/L: 4.92 ± 1.12), 11 (15.94%) had sufficient (20–30 nm/L: 24.71 ± 3.40), and 4 (5.80%) had optimal values (>30 nm/L: 47.17 ± 16.85) (Table 2) (Figure 1).

No other significant intergroup differences (familial psychiatric history, specific psychopharmacologic treatment, medical comorbidity, affective episodes, diagnosis, gender, etc.) were noted (Table 3 and Table 4).

The same was true when comparing vitamin D according to diagnoses, the polarity of the episode, psychotropic drugs, type of onset, family history for psychiatric disorders, smoking, comorbidities, presence or absence of psychotic symptoms, abuse substances, or neurodevelopmental traits (Table 3 and Table 4).

## 4. Discussion

The present study aimed at evaluating vitamin D levels, in comparison with normative values, in a group of adult patients suffering from BDs and hospitalized for a mood episode (manic, hypomanic, depressed, or mixed). All subjects were well characterized from the socio-demographic and clinical points of view. In addition, we explored the possible correlations between vitamin D values and the psychopathological characteristics of the patients.

The results showed that the mean vitamin D levels of the patients were significantly lower (14.58 ± 11.27 nmol/L) than the normative values (>30 nmol/L). Eleven patients had sufficient values and only 4 had optimal, while 19 showed insufficient, 18 critical, and 17 severely critical levels.

In our sample, no differences in vitamin D levels were noted between men and women or when individual and clinical features were considered and analyzed.

Taken together, these data indicate that a large percentage (almost 80%) of patients affected by BDs showed decreased levels of vitamin D, and they add further information to the literature on this topic, as available data are meager and controversial. Previously, vitamin D deficiency has been reported in acute manic episodes [42]. Specifically, patients with acute manic episodes had significantly lower vitamin D serum concentrations than healthy controls, but the remission group’s serum concentrations did not differ significantly from healthy controls or acute manic episode patients. Furthermore, a correlation between vitamin D levels, YMRS scores, and CGI scores was observed. Such correlations with symptom severity were not detected in our sample of bipolar patients, as we noted no association between levels of vitamin D and the polarity of the episode. The controversial findings might be explained by different factors: heterogeneity of the patients and diagnostic criteria, small samples, and different methods to measure vitamin D and define its thresholds.

It has been hypothesized that vitamin D deficiency and acute manic episodes may be related according to a dysregulation of the glutamate system [59]. Specifically, glutamatergic and GABAergic abnormalities observed in BD would result from a stress-induced cortisol increase enhancing the intrasynaptic glutamate levels. These lead to the overactivation of postsynaptic N-methyl-D-aspartate (NMDA) glutamate receptors and subsequent increased intracellular Ca^2+^ concentration, typical of mania. Interestingly, vitamin D has been associated with reductions in Ca^2+^ levels [60]. Even the opposite hypothesis can be speculated, that is to say, a decrease in vitamin D may be related to an increase in Ca^2+^ concentration [57] that can damage the GABAergic system, resulting in manic symptoms. In line with this hypothesis, an 8-week vitamin D3 supplementation (a daily dose of 2000IU) significantly decreased YMRS scores [47].

Recently, Cereda et al. (2021), while reviewing the literature, underlined the existence of a possible correlation between vitamin D levels and depressive or manic symptoms. Unfortunately, this finding cannot be considered a biomarker specific to BDs, as it has been also noted in schizophrenia, MDD, and OCD [33,45]. Similarly, Boerman et al. (2016) found that vitamin D deficiency is 4.7 times more common in patients with BDs, schizophrenia, or schizoaffective disorder than in the Dutch white general population of maintaining appropriate levels and providing regular assessments of the vitamin [43].

In MDD, controversies still exist on the real role of vitamin D in this condition, especially because the available evidence is limited to a few heterogeneous studies. A link between depressive symptoms and lower vitamin D levels was noted in a sample of young adults [36] and 196 hospitalized depressed patients [37]. In a sample consisting of participants (aged 18–65 years) with a current (N = 1102) or remitted (N = 790) depressive disorder (major depressive disorder, dysthymia) and healthy controls (N = 494), it was found deficient or insufficient serum levels of vitamin D in 33.6% of patients, after adjusting for sociodemographics, sunlight, urbanization, lifestyle, and health. Lower vitamin D levels were found in participants with current depression, particularly in those with the most severe symptoms with an inverse correlation, suggesting a dose-response gradient [38].

These findings were confirmed in another study including more than 2070 elderly participants (aged ≥ 65 years) [39]. In this case, long-standing physical health issues can affect both a person’s risk for depression and access to sun exposure. However, after adjusting for this last factor, the study revealed only modest confounding effects. The direction of the causal link is perhaps the most important consideration and cannot be inferred from a cross-sectional design. It is possible that the depressive states were a cause, rather than a consequence, of vitamin D deficiency, although if so, one would expect the association with depression to be similar in extent with any relative rather than with limited vitamin D deficiency at the lowest 10% levels. Furthermore, if depression caused vitamin D deficiency due to lack of sunlight exposure, then the association between vitamin D levels and contemporary depressive symptoms would be more robust in the summer months when levels are highest: however, this expectation was not fulfilled in that sample [39]. Manzanos et al. (2022) speculated that one of the possible mechanisms through which vitamin D might affect brain function and mood is the activity of different neurotransmitters, such as serotonin (5-HT), dopamine (DA), and noradrenaline (NA) [40].

Vitamin D would appear to play an important role in managing synthesis and maintenance of normal levels of 5-HT through 5-HT tryptophan hydroxylase 2 promoter activation while repressing 5-HT tryptophan hydroxylase 1 expression; this would indicate that vitamin D insufficiency would alter 5-HT production, with the resulting alteration in mood. There is also preclinical and clinical evidence of vitamin D’s capacity to increase brain levels of DA and NA. It would seem that it also triggers its effects by activating tyrosine hydroxylase, an enzyme with a DA synthesis-limiting function. Furthermore, vitamin D supplementation is known to increase DA and NA levels in different areas and circuits linked to mood. In addition, by controlling the expression of responsible genes, vitamin D participates in maintaining the homeostasis of Ca^2+^ and oxidative stress reactive oxygen species [ROS]), an essential balance for maintaining healthy and functional neurons. While hypovitaminosis D increases Ca^2+^ and ROS by altering neural function, maintaining normal vitamin D levels leading to a reduction of Ca^2+^ might be another plausible mechanism for preventing or improving depression [40].

The eventual efficacy of vitamin D addition as a booster of other psychotropic drugs is controversial. Adjunctive vitamin D supplementation seemed to be beneficial as a single parental dose (300,000 IU) not only for depressive symptoms but also for the overall quality of life and illness severity [50]. In addition, a systematic review highlighted that vitamin D supplementation was associated with a reduction in both depressive and manic symptoms [45]. The role of gender in response to the treatment has also been investigated, with women showing a more significant improvement than men in their depressive symptoms following a three-month supplementation with 50,000 I.U. vitamin D [61].

The efficacy of vitamin D addition may also be different according to other variables that should be taken into account, such as the presence of other clinical features that flank the merely depressive symptoms. Indeed, recent research might support these statements, as an amelioration of anxiety symptoms was noted in patients who were not depressed but had vitamin D deficiency [62]. Again, given the relevant prevalence of vitamin D deficiency in a large part of elderly subjects, any medical comorbidity, quite common in these patients, might represent another variable affecting the response to vitamin D supplementation. It is noteworthy that an improvement in depressive symptoms was recently demonstrated in a group of patients affected by type 2 diabetes. Moreover, the improvement of several metabolic parameters (i.e., insulin, HbA1c) was also reported, as vitamin D may facilitate insulin release from pancreatic beta-cells [63]. As already mentioned, as vitamin D deficiency is common amongst children and adolescents, it should be noted that a study carried out on 54 Swedish depressed adolescents reported improvement in the well-being of specific symptoms after supplementation of vitamin D for three months [64].

Interestingly, vitamin D supplementation in combination with fluoxetine seems to be more effective than fluoxetine alone or plus placebo in reducing depressive symptoms [65]. In three small pilot studies, vitamin D supplementation showed a positive effect on well-being, and the symptoms of depression were improved when high doses of vitamin D (≥100 μg D3 daily) were given for 1 to 3 months [66,67]. Bakhtiari-Dovvombaygi et al. (2021) also reported that the anti-inflammatory and antioxidant effects displayed by pretreatment with vitamin D3 (10,000 IU/kg for 28 days) in male rats would be the basis of the ability of this vitamin to abrogate anxiety- and depressive-like behaviors induced by chronic unpredictable mild stress (CUMS) in the elevated plus-maze and forced swimming tests. Indeed, these protective effects of vitamin D were accompanied by a decrease in cortical malondialdehyde and IL-6 levels, as well as an increase in total thiol levels and enhanced SOD and catalase activity [68].

Besides, the spectra of vitamin D activities have been further broadened by the discovery of both the so-called nuclear vitamin D receptors (VDRs), which are widely distributed in the human body, and the vitamin D response elements (VDRE) present in the promoter regions of several genes [69,70]. Vitamin D receptors are supposed to play a critical role in the course of embryonic brain development, given the evidence of their expression in different brain areas, including the neurons producing dopamine in the substantia nigra [71] and in microglia, astrocytes, and oligodendrocytes [28,72,73]. Overall, the distribution of VDRs in the brain would provide an explanation of the multiple roles of vitamin D in several fundamental brain processes, such as axogenesis [24], promotion of several neurotrophic factors (i.e., NGF and BDNF) [74,75], and neuroprotection by inhibiting nitric oxide (NO) production [28], all processes that result to be altered in MDs [11]. Interestingly, mutation of the VDR gene may lead to abnormal receptor proteins that are also implicated in the regulation of glucocorticoid signalling, a process altered in MDs [76].

According to further data, the neuroprotective effect of vitamin D on the brain might be also due to its ability to lower plasma C-reactive protein in patients with psychiatric disorders and to modulate inflammation by suppressing pro-inflammatory cytokines. There is evidence of vitamin D exerting significant anti-inflammatory effects by activating the Th2 anti-inflammatory branch and decreasing pro-inflammatory Th1 cytokines. Through this pathway, vitamin D might decrease the associated pro-inflammatory state widely reported in some patients with depression [66].

### Limitations

The present study suffers from a few limitations to be acknowledged. First, the majority of our patients were taking one or more psychotropic drugs to manage their clinical picture, and only seven were drug-free. It should be underlined that it is uncommon to recruit untreated bipolar inpatients, and, in any case, we did not find any difference between treated or drug-free patients or between patients taking one or more drugs. Second, there was a preponderance of women than men, however, this is almost the rule in samples of bipolar patients; in addition, no sex-related differences were noted in the clinical parameters or vitamin D levels. Third, all patients were recruited in a short period of time (March–June 2021) to reduce the impact of differential exposure to the light on vitamin D levels. Again, it should be underlined that they belonged to the same geographical area, with similar life habits and Mediterranean diet. Third, although the numerosity of our sample was consistent as compared with those of similar studies and composed of both men and women within a small age range, maybe it was not sufficient to ascertain intergroup differences in the biomarkers in patients at different phases of the illness. Another limitation was that we compared the vitamin D levels with the normative values rather than with control subjects, as we are planning to do in future studies.

## 5. Conclusions

Our study, albeit preliminary, supports the evidence of decreased vitamin D in a large sample of BDs. This is an intriguing finding that obviously requires to be substantiated in future studies in order to deepen the possible role of vitamin D in this and, possibly, other neuropsychiatric disorders. Indeed, it is now evident that it regulates and modulates a multiplicity of brain and bodily functions that may be involved in psychopathological disorders. Specifically, vitamin D insufficiency would interfere with the function of neurons and neural circuits linked to mood fluctuations. It indicates plausible pathways for understanding that hypovitaminosis D alters brain function and, on a secondary basis, causes mood instability. It also explains plausible mechanisms for the preventive effects of prior suitable levels and the clinical improvement brought by adding vitamin D to conventional treatment for MDs.

Further and controlled studies will be necessary to assess the potential benefits of vitamin D per se or as an augmentation agent, at least in those patients showing decreased levels. In any case, taken together, although drug regulatory agencies in different countries may warn against excessive prescriptions of vitamin D, these findings and the literature suggest the importance of regular assessments of vitamin D and its supplementation when needed. 

## Figures and Tables

**Table 1:** Demographic and clinical data of bipolar inpatients.

Total Patients (69: 29 M; 40 F)	Age: 45.09 ± 14.42
Diagnoses	
BDI (27: 11 M, 16 F)	
BDII (15: 4 M, 11 F)	
Schizoaffective (16: 11 M, 5 F)	
MDD (11: 3 M, 8 F)	
Mood episode	
Manic (6: 4 M; 2 F)	
Hypomanic (7: 3 M, 4 F)	
Depressive (36: 12 M; 24 F)	
Mixed (20: 10 M, 10 F)	

Age: years, mean ± SD; M = male; F = female; BDI = Bipolar disorder of type 1; BDII = Bipolar disorder of type 2; MDD = Major Depressive Disorder.

**Table 2:** Vitamin D levels (nmol/L, mean ± SD) of bipolar inpatients.

Subjects	Values	N
Total sample	14.58 ± 11.27	69
Women	14.48 ± 10.24	40
Men	14.72 ± 12.74	29
Insufficiency	15.70 ± 2.17	19
Critical deficiency	9.10 ± 1.49	18
Severe critical deficiency	4.92 ± 1.12	17
Sufficient levels	24.71 ± 3.40	11
Optimal values	47.17 ± 16.85	4

**Table 3:** Vitamin D comparisons according to diagnosis, episode type, second diagnosis type, and pharmacological treatment association using Kruskal-Wallis test.

	H	df	*p*
Diagnosis	1.010	3	0.799
Episode Type	3.083	3	0.379
Second Diagnosis Type	1.275	3	0.735
Pharmacological treatment association *	9.046	8	0.338

Legend. df = variance degree of freedom. *p* < 0.05 significant. * = groups compared: mood stabilizer alone/double mood stabilizers alone/antidepressants alone/antipsychotics alone/double antipsychotics alone/mood stabilizer + antidep/mood stabilizer + antipsychotic/mood stabilizer + antid. + antipsychotic/mood stabilizer + antidepressant + benzodiazepines/mood stab + antidep. + antipsychotic + benzodiazepines.

**Table 4:** Vitamin D comparisons according to different variables (reported below) using independent sample Mann-Whitney test.

	U	Z	*p*
Gender	553,000	−0.87	0.930
Psychosis	574,500	−0.18	0.985
Acute onset	370,500	−0.399	0.690
Bipolar condition	493,000	−0.835	0.404
Psychiatric comorbidity	472,000	−1.097	0.273
Lithium	365,000	−0.970	0.332
Tricyclics	17,500	−1.761	0.078
Mood stabilizer	345,000	−1.143	0.253
Double mood stabilizer	343,500	−0.799	0.424
Antipsychotic	159,500	−0.573	0.567
Double antipsychotic	374,000	−1.057	0.291
Antidepressant	474,000	−0.997	0.319
Double antidepressant	120,500	−0.195	0.845
Medical comorbidity	488,500	−0.955	0.340
Smoking	556,000	−0.256	0.806
Psychiatric family loading	321,500	−0.233	0.816
Neurodevelopmental traits	256,500	−0.580	0.562
Suicidal family loading	171,500	−1.304	0.192
Substance Users	325,500	−1.355	0.175

Legend. *p* < 0.05 significant.

## Data Availability

All data generated or analysed during this study are included in this published article.
